# Allergy-compatible symptoms among federated swimmers in Portugal: a cross-sectional study using the AQUA® questionnaire

**DOI:** 10.3389/fpubh.2025.1731628

**Published:** 2026-01-12

**Authors:** Miguel Ramos, Henrique P. Neiva, Olga Lourenço

**Affiliations:** 1 Faculty of Health Sciences, University of Beira Interior, Covilhã, Portugal; 2 Department of Sport Sciences, University of Beira Interior, Covilhã, Portugal; 3 Research Centre in Sport Sciences, Health Sciences and Human Development (CIDESD), Covilhã, Portugal; 4 Department of Medical Sciences, Faculty of Health Sciences, RISE-Health, University of Beira Interior, Covilhã, Portugal

**Keywords:** swimming, athletes, allergic symptoms, asthma, indoor air quality, chloramines, AQUA questionnaire, cross-sectional study

## Abstract

Swimmers are chronically exposed to water disinfection by-products (commonly from chlorination) and to indoor air conditions that may aggravate allergic and respiratory symptoms. Evidence is robust for elite cohorts, but competitive swimmers outside Olympic settings remain understudied. We conducted a cross-sectional, anonymous online survey among Portuguese competitive swimmers, officially registered with the national federation, aged 16 years or older (April–May 2025). The validated Allergy Questionnaire for Athletes (AQUA^®^; 21 items; positive ≥5 points) screened for allergy-compatible symptoms. Associations with demographics, training exposure, and pool environment were examined using descriptive statistics and bivariate tests (*α* = 0.05); exploratory logistic regression assessed independent predictors. Of 100 respondents, 95 valid questionnaires were analyzed. The prevalence of AQUA-positive screening was 81.1% (*n* = 77/95). Upper-airway complaints were most frequent (e.g., nasal obstruction 63.2%; rhinorrhea 61.1%), followed by cough 55.8% and ocular pruritus (51.6%). AQUA positivity was higher in women vs. men (88.7% vs. 71.4%, *p* = 0.036). Younger age (16–25 years) and ≥7 weekly training sessions were also associated with higher positivity (*p* = 0.011 and *p* = 0.012, respectively). A temperature gradient was evident (≤25 °C 50.0%; 25–27 °C 86.4%; 28–30 °C 83.3%; *p* = 0.013). Prior medical diagnosis of allergic disease and use of anti-allergic medication was strongly associated with AQUA positivity (*p* < 0.001). In multivariable analysis, female sex, younger age, ≥7 sessions/week, and pool water temperature >25 °C remained independent predictors. Allergy-compatible symptoms were common among competitive swimmers, with environmental and training-related correlations. Findings support routine symptom screening (AQUA) in clubs alongside attention to pool management (temperature, ventilation). Larger studies with clinical confirmation and objective environmental monitoring are warranted.

## 1 Introduction

Allergic diseases are among the leading causes of chronic morbidity worldwide, affecting over a third of the population and encompassing conditions such as allergic rhinitis, bronchial asthma, atopic dermatitis, urticaria, and allergic conjunctivitis, largely mediated by type I hypersensitivity (1). Their rising prevalence imposes substantial burdens on quality of life, productivity, healthcare costs, and (for athletes) training and performance (2).

In sport, allergic disease deserves particular attention in disciplines with high cardiorespiratory demand and indoor practice, where suboptimal ventilation and exposure to chemical agents can exacerbate respiratory symptoms (3). Multiple studies consistently report higher rates of respiratory complaints and diagnosed allergic disease in swimmers compared with athletes from land-based sports (4, 5). Further evidence suggests that swimmers may present more symptoms and a higher use of medication than endurance athletes in disciplines such as athletics or cycling (5).

Swimming entails repetitive contact with chlorinated water and (in indoor venues) volatile disinfection byproducts, notably chloramines, which act as airway irritants and can increase epithelial permeability and airway inflammation. Competitive swimmers, including elite cohorts, consistently show elevated respiratory morbidity, with asthma prevalences among the highest across sports (often >40%) and increased allergic and rhinitis symptoms (4, 6–12). Mechanistic and epidemiological studies link these findings to prolonged exposure to water disinfection by-products, such as chloramines and trichloroisocyanuric acid, particularly in heated indoor pools (13–15). Other studies show signs of airway inflammation even in the absence of a formal asthma diagnosis, underscoring the physiopathological impact of aquatic exposure on respiratory function in this athletic population (8–12).

Experimental and field studies have clarified the biological pathway through which these exposures exert their effects. Volatile chloramines, especially trichloramine, can disrupt epithelial tight-junctions, increase airway epithelial permeability, induce oxidative and nitrosamine stress, and activate inflammatory cascades that facilitate immune sensitization to inhaled allergens (16–18). When volatilization is enhanced by higher water temperatures and ventilation is suboptimal, airborne concentrations may rise substantially, increasing inhalational dose during exercise (19). Repeated exposure under high ventilatory demand may contribute not only to acute irritation but also to chronic epithelial remodeling, which has been documented among competitive swimmers (20, 21). These mechanisms help contextualize the elevated burden of allergy-compatible symptoms observed in aquatic athletes.

Despite this, most evidence is derived from elite or Olympic cohorts and/or resource-intensive physiological testing that is less feasible for population-level surveillance (3, 4, 22). Few studies have incorporated standardized, sport-adapted screening tools suitable for broad application (23, 24). Comparative research with other sports further supports a specific association between swimming, bronchial hyperreactivity, and allergic symptoms—often more pronounced in younger athletes. It must also be acknowledged that common symptoms such as cough, sneezing, ocular pruritus, or wheeze can arise from non-allergic conditions, including recurrent viral infections, active or passive smoking, or environmental pollution; household ventilation and the use of cleaning chemicals may constitute relevant, unmeasured confounders in several studies (25). These gaps underscore the need for pragmatic, validated screening approaches in real-world settings.

Given these gaps, practical tools such as standardized clinical questionnaires are particularly valuable for large-scale, sport-relevant screening. The Allergy Questionnaire for Athletes (AQUA) is a 21-item tool assessing respiratory, ocular, and cutaneous symptoms; scores ≥5 indicate allergy-compatible manifestations. While validated across languages and contexts, evidence has mainly focused on elite swimmers. This study uses AQUA to characterize the prevalence and correlations of allergy-compatible symptoms in non-elite, federated swimmers, addressing this gap. Therefore, the current study aimed to estimate the prevalence of AQUA-positive screening among competitive swimmers aged ≥16 years and to examine associations between symptoms and demographic, training, and pool-environment variables in Portugal. Secondary aims were to describe self-reported diagnoses, medication use, and to explore independent predictors of AQUA positivity.

Based on existing evidence, we hypothesized that, although federated swimmers experience lower cumulative exposure than elite swimmers, their regular training in chlorinated pools would still result in a prevalence of allergy-compatible symptoms higher than that observed in the general population. Furthermore, we hypothesized that environmental conditions (such as water temperature and perceived air quality) and individual characteristics (including age, sex, and weekly training frequency) would be significantly associated with AQUA positivity. Understanding these relationships may support more informed decisions by coaches, clinicians, and pool operators regarding early symptom recognition, appropriate referral, and environmental management strategies.

## 2 Materials and methods

### 2.1 Study design and setting

We conducted an observational cross-sectional study via an anonymous online questionnaire between April and May 2025 (two months). The Portuguese Swimming Federation centrally disseminated the survey link to affiliated clubs for onward distribution to registered swimmers. Participation was voluntary; the platform required complete responses before submission to prevent partial entries. Electronic informed consent was obtained before questionnaire access. To ensure data integrity, the survey platform prevented duplicate submissions from the same device/session, and timestamps were screened to identify potential repeated entries. All complete questionnaires were manually checked for internal consistency; no duplicate or inconsistent responses required exclusion.

### 2.2 Participants

Eligible participants were male and female swimmers aged ≥16 years, registered in the Portuguese Swimming Federation during the 2024–2025 competitive season. Inclusion criteria were: (i) current federated status; (ii) age ≥16 years; (iii) informed consent; and (iv) complete questionnaire submission. Exclusion criteria comprised: (i) age <16 years; (ii) incomplete questionnaires; or (iii) refusal to provide informed consent.

A total of 100 responses were received, of which 95 fulfilled the inclusion criteria and were analyzed.

### 2.3 Instrument

The tool was the Allergy Questionnaire for Athletes (AQUA), developed by Bonini et al. (23) and validated in multiple languages and sporting contexts, including Portuguese athletes (24). The instrument comprises 21 items addressing respiratory, ocular, and cutaneous symptoms, medical history, and medication use. Each affirmative response contributes one point, and the sum of positive answers yields a total score. Values ≥5 denote a positive screen (AQUA-positive), suggestive of allergy-compatible manifestations. This threshold was established during validation studies, showing high sensitivity for detecting athletes with clinically confirmed allergies while maintaining acceptable specificity (23). Consistent with its intended use, AQUA functions as a screening tool for allergy-compatible symptoms rather than as a diagnostic instrument, and positive results require clinical evaluation for confirmation.

### 2.4 Variables and outcomes

Collected variables included:

- Demographic: sex, age, region of residence.
- Training-related: years federated, weekly training frequency, self-reported intensity.
- Environmental: pool setting (indoor/outdoor), water temperature, perceived air quality, use of respiratory protective equipment.
- Clinical: symptom profile, prior allergic diagnosis, medication use (antihistamines, corticosteroids, bronchodilators, other).
- Infectious history: respiratory infections in the previous year.

The primary outcome was the prevalence of AQUA-positive participants. Secondary outcomes included the distribution of reported symptoms, frequency of medical diagnosis, medication use, and associations between an AQUA-positive screen and demographic, training, or environmental variables.

Environmental exposures were self-reported by swimmers based on their habitual training environment. Water temperature and air quality were not instrumentally measured, and no standardized time-of-day measurement protocol was used. These values, therefore, represent perceived conditions rather than objective environmental sampling.

### 2.5 Sample size

Sample size was estimated to be *a priori* for a single-proportion outcome (prevalence of AQUA-positive screening). The expected prevalence (*p* = 0.27) was chosen for three reasons: (i) it lies within the 20–30% range reported in population-based surveys of allergic disease in young adults, (ii) it is substantially lower than figures described for elite swimmers, better reflecting the non-elite federated context targeted here, and (iii) a mid-to-lower estimate avoids inflating the required sample while retaining adequate precision for this population. Using Z = 1.96 (95% confidence) and an absolute precision of 9% (d = 0.09), the required sample was *n* = 96 according to *n* = Z^2^·p(1–p)/d^2^. The final dataset comprised 95 valid questionnaires, closely matching this target.

### 2.6 Statistical analysis

Descriptive statistics summarized the sample (means, standard deviations, frequencies, and percentages). Associations between AQUA-positive status and categorical variables were tested with chi-square tests (χ^2^) or Fisher’s exact test when appropriate. Independent-samples t-tests were applied to continuous variables. Normality of continuous variables was assessed using the Shapiro–Wilk test and visual inspection of Q-Q plots before applying parametric tests. Effect sizes were calculated to complement bivariate analyses. For categorical variables, association strength was evaluated using Cramer’s V. Values of approximately 0.10, 0.30 and 0.50 were interpreted as small, medium and large associations, respectively. A significant threshold of *p* < 0.05 was adopted. Logistic regression analyses were explored to identify independent predictors of AQUA-positive status. Before multivariable logistic regression, potential multicollinearity among predictors was evaluated using the Variance Inflation Factor (VIF), with all values <2, indicating acceptable independence between variables. Reference categories for regression models were defined *a priori*: male sex, age >25 years, <7 weekly training sessions, and water temperature ≤25 °C. The final multivariable output, including adjusted odds ratios, confidence intervals, reference categories, and VIF values, is presented in the updated Supplementary Table S1. All analyses were performed using IBM SPSS Statistics, version 29 (IBM Corp., Armonk, NY, United States).

### 2.7 Ethics

The study was conducted in accordance with the Declaration of Helsinki. Ethical approval was obtained from the Ethics Committee of the University of Beira Interior (CE-UBI-Pj-2024-089-ID2769). All participants provided informed consent prior to participation. Data was anonymized and handled according to European Union General Data Protection Regulation (GDPR) standards.

## 3 Results

### 3.1 Participant characteristics

**Figure 1:**
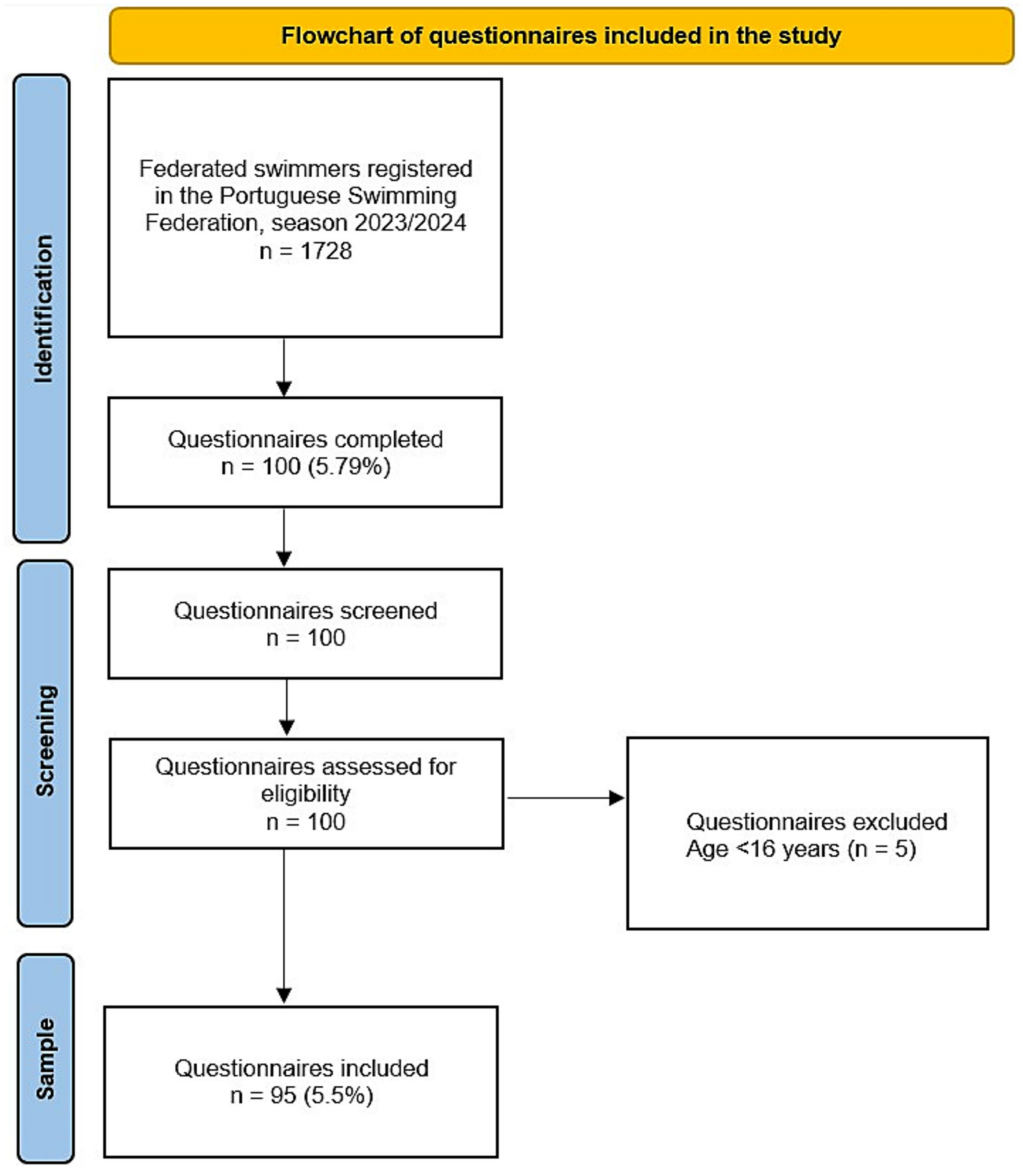
Flowchart of questionnaires included in the study.

A total of 100 questionnaires were received, of which 95 fulfilled the inclusion criteria and were analyzed (Figure 1). Participants had a mean age of 20.9 ± 6.3 years (range: 16–50), and 55.8% (*n* = 53) were female. The mean duration of federated swimming was 8.3 ± 6.3 years. Most swimmers (78.9%) reported training ≥4 sessions per week, with a mean of 6.5 ± 2.5 weekly sessions. Regarding self-reported training intensity, 57.9% described it as moderate and 42.1% as high. Most trained predominantly in indoor pools (81.1%), and 68.4% reported water temperatures above 25 °C. Perceived air quality was rated as satisfactory by 72.6% of respondents. Table 1 summarizes the demographic and training characteristics.

**Table 1:** Reported symptoms among federated swimmers (AQUA items).

Symptom	Number of swimmers (%)
Cough, shortness of breath and/or throat itching	55 (58%)
Sneezing and nasal itching	38 (40%)
Wheezing and/or chest tightness	33 (35%)
Red eyes, itching and/or tearing	25 (26%)

### 3.2 Prevalence of allergy-related symptoms

AQUA-positive screening prevalence was 81.1% (*n* = 77/95). Upper-airway symptoms predominated: nasal obstruction (63.2%), rhinorrhea (61.1%), sneezing (57.9%), and nasal pruritus (53.7%). Ocular symptoms included pruritus (51.6%) and tearing (47.4%). Lower-airway symptoms were also frequent: cough (55.8%), wheeze (34.7%), and dyspnea (27.4%). Cutaneous manifestations were less common (urticaria 13.7%, eczema 9.5%); gastrointestinal complaints were rare (3.2%). Table 2 details symptom frequencies.

**Table 2:** Self-reported allergic diseases among federated swimmers.

Allergic disease	Number of participants (%)
Asthma	8 (8.4%)
Allergic rhinitis	14 (14.7%)
Atopic dermatitis (Eczema)	6 (6.3%)
Urticaria	0
Allergic conjunctivitis	1 (1.1%)
Anaphylaxis	1 (1.1%)
Food allergy	5 (5.3%)
Drug allergy	4 (4.2%)
Insect allergy	1 (1.1%)

### 3.3 Medical diagnoses and medications use

**Figure 2:**
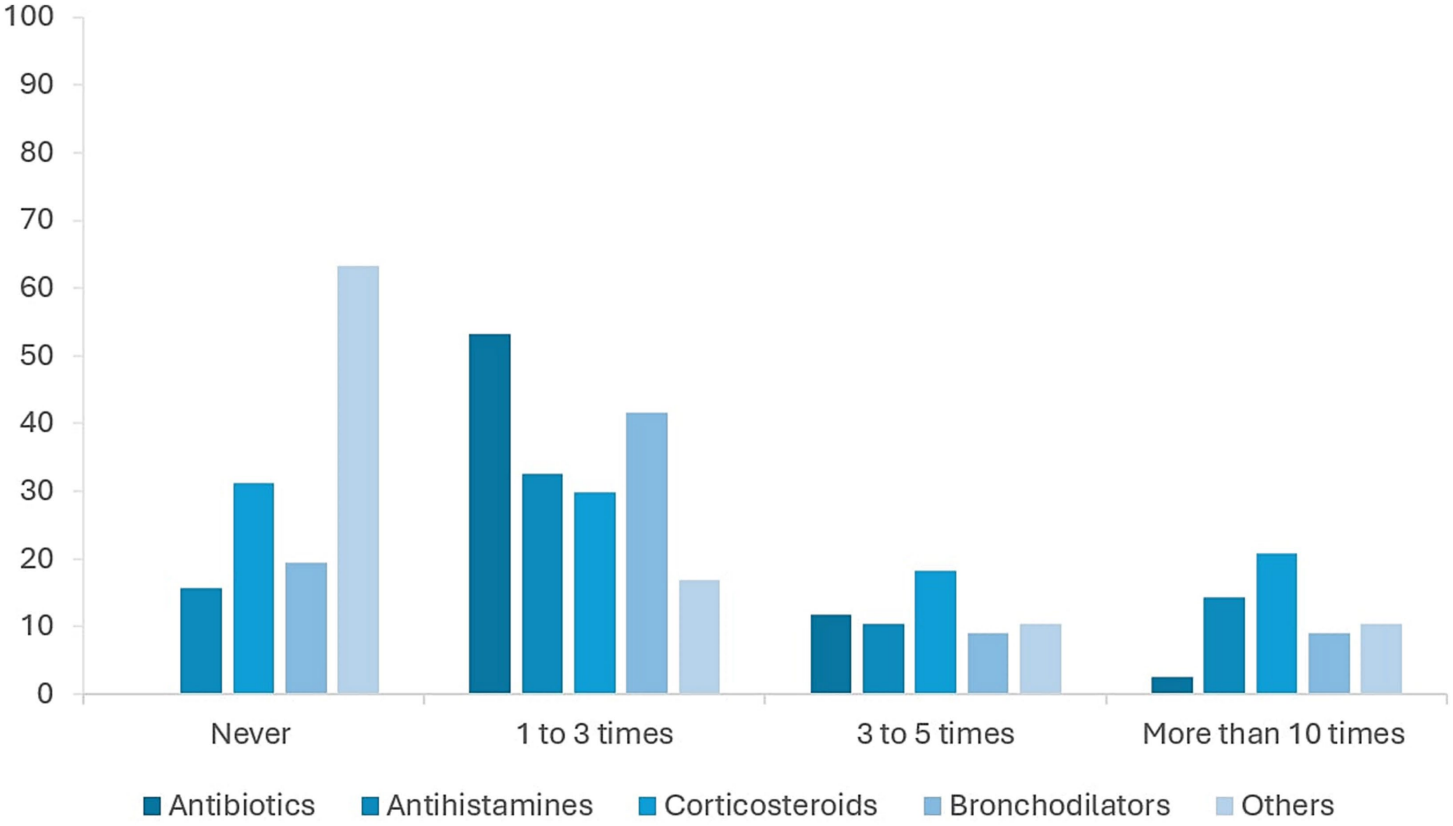
Frequency of medication use among federated swimmers, by drug class. Bars represent the proportion of AQUA positive swimmers reporting use of each medication category: antihistamines, corticosteroids, bronchodilators, and other anti-allergic drugs. Percentages are based on the total number of AQUA-positive respondents (*n* = 77).

A total of 40.0% (*n* = 38) of swimmers reported a prior medical diagnosis of allergic rhinitis, 25.3% (*n* = 24) of asthma, and 15.8% (*n* = 15) of other allergic conditions. Among AQUA-positive athletes, 59.7% had at least one formal medical diagnosis. Medication use was frequent: antihistamines were the most common (34.2%), followed by corticosteroids (22.4%), bronchodilators (18.4%), and other anti-allergic drugs (12.0%). Figure 2 shows medication distribution.

### 3.4 Associations with demographic and training variables

Female swimmers were more frequently AQUA-positive than males (88.7% vs. 71.4%, *p* = 0.036). Age also showed a significant association, with 88.9% of participants aged 16–25 years being AQUA-positive compared with 64.3% of those >25 years (*p* = 0.011). Training frequency was positively associated with AQUA status: 89.2% of swimmers training ≥7 sessions/week were positive, versus 67.6% among those training fewer sessions (*p* = 0.012). Duration of federated swimming and self-reported intensity showed no significant associations. Table 3 summarizes these associations.

**Table 3:** Association between environmental factors (water temperature, air quality) and AQUA positivity.

Environmental factor	Category	Allergic (*n* = 77)	Non-allergic (*n* = 18)	Total (*n*)	95% CI	*p*-value
Water temperature	<25°C	50.0% (6)	50.0% (6)	100% (12)	[0.751–14.090]	0.0127*
	25–27 °C	86.4% (51)	13.6% (8)	100% (59)		
	28–30 °C	83.3% (20)	16.7% (4)	100% (24)		
Air quality	Very good	80.0% (24)	20.0% (6)	100% (30)		0.157
	Good	73.7% (28)	26.3% (10)	100% (38)		
	Fair/Poor	92.6% (25)	7.4% (2)	100% (27)		

*Means that result is statistically significant.

### 3.5 Associations with environmental and clinical variables

Environmental conditions played a role in symptom burden. Water temperature demonstrated a significant gradient: ≤25 °C, 50.0%; 25–27 °C, 86.4%; 28–30 °C, 83.3% (*p* = 0.013). Perceived air quality showed a non-significant tendency, with lower prevalence among those reporting “good” air quality. Pool setting (indoor vs. outdoor) and use of respiratory protective equipment were not significantly associated with AQUA status. Clinically, both prior medical diagnosis and use of anti-allergic medication were strongly associated with *AQUA-positive* status (*p* < 0.001). Recurrent respiratory infections were reported by 41.1% of swimmers, with no significant difference between groups. Full results are provided in Table 4.

**Table 4:** Association between clinical variables (previous diagnosis, anti-allergic medication, family history) and AQUA positivity.

Clinical variable	Category	Allergic (*n* = 77)	Non-allergic (*n* = 18)	Total (*n*)	95% CI	*p*-value
Previous diagnosis	Yes	100.0% (28)	0.0% (0)	100% (28)		
	No	73.1% (49)	26.9% (18)	100% (67)		
Anti-allergic medication	Yes	97.7% (42)	2.3% (1)	100% (43)		<0.001
	No	67.3% (35)	32.7% (17)	100% (52)		
Family history of allergy	Yes	88.4% (38)	11.6% (5)	100% (43)	[0.208–8.845]	0.089
	No	74.5% (38)	25.5% (13)	100% (51)		

### 3.6 Exploratory regression analysis

In the multivariate logistic regression, female sex (OR = 3.15; 95% CI 1.05–9.45), age 16–25 years (OR = 4.32; 95% CI 1.21–15.38), ≥7 weekly training sessions (OR = 3.76; 95% CI 1.15–12.27), and pool water temperature >25 °C (OR = 5.41; 95% CI 1.32–22.10) remained independent predictors of an AQUA-positive screen. Model calibration by Hosmer–Lemeshow test indicated adequate fit (χ^2^(6) = 0.885, *p* = 0.990).

These findings indicate that younger swimmers, females, those training more frequently, and those exposed to warmer pool water have substantially increased odds of screening positive on the AQUA questionnaire, even after accounting for other demographic, environmental, and training variables.

A complete summary of regression coefficients, odds ratios, 95% confidence intervals, reference categories, multicollinearity diagnostics, and model statistics is presented in Supplementary Tables S1–S3. A visual summary of the model using a forest plot of adjusted odds ratios and corresponding confidence intervals is provided in Supplementary Figure S1.

## 4 Discussion

This study aimed to characterize the prevalence and correlations of allergy-related symptoms in a national cohort of non-elite competitive swimmers using the validated AQUA questionnaire. We found that four in five swimmers screened AQUA-positive, a prevalence markedly higher than general-population estimates and consistent with international evidence documenting increased respiratory morbidity among swimmers. These findings demonstrate that the burden of allergic and respiratory symptoms extends beyond Olympic and elite cohorts, underscoring the relevance of systematic surveillance in federated contexts.

Competitive swimmers—including elite cohorts—have consistently shown elevated respiratory morbidity, with asthma prevalences among the highest across sports (often >40%) and increased allergic and rhinitis symptoms (4, 6–12). The symptom pattern in our cohort—dominant upper-airway involvement with frequent cough and ocular symptoms—is consistent with irritant effects from chlorinated water and volatile by-products, especially in heated, indoor pools (13–15). Airway inflammation has been observed even without a formal asthma diagnosis, underscoring the physiopathological impact of aquatic exposure on respiratory function in this athletic population (8–12).

Volatile chloramines, particularly trichloramine, have been shown to disrupt epithelial tight junctions, increase airway permeability, and activate inflammatory pathways that promote immune sensitization and chronic airway remodeling (16, 20, 26). These mechanisms are biologically plausible and help explain the elevated burden of symptoms observed in swimmers.

Chronic airway irritation may also impair respiratory efficiency and oxygen uptake during exercise. Studies using exhaled breath analysis and metabolomic profiling have demonstrated altered respiratory parameters in swimmers exposed to chlorinated environments, suggesting that repeated exposure may compromise ventilatory performance and aerobic capacity (17, 27).

Significant associations were observed with sex, age, training frequency, and water temperature. Female swimmers presented a higher prevalence of AQUA positivity, in agreement with studies suggesting sex-based susceptibility to allergic disease (28, 29). Younger age (16–25 years) was also a risk factor, consistent with data showing that allergy incidence peaks in adolescence and early adulthood (1, 30). Training frequency emerged as an independent predictor, supporting the role of cumulative exposure to chlorinated water and air by-products in symptom development (31, 32). The temperature gradient (≥25 °C vs. ≤25 °C) is biologically plausible given the favored formation/release of chlorination by-products at warmer temperatures (16, 26). Several variables—including perceived air quality, pool setting, and years of federated practice—were not significantly associated with AQUA-positive screening. These non-significant findings suggest that, within this non-elite swimming population, symptom burden may be more strongly driven by individual susceptibility and training volume than by broad contextual or exposure-duration factors. Nevertheless, the absence of significance does not exclude potential subtle effects that might become apparent with objective environmental monitoring or larger samples.

Contrary to expectations, no significant associations were found for years federated, self-reported training intensity, pool setting (indoor vs. outdoor), or use of respiratory protective equipment. Several explanations are plausible. First, exposure misclassification is likely: years federated, and a single self-rated intensity item are imprecise surrogates of cumulative dose (they do not capture session duration, rest intervals, or ventilation patterns), which can attenuate true effects toward the null. Second, limited between-group contrast may have reduced power—most swimmers trained predominantly indoors (≈80%), leaving little variability to detect setting-related differences, while protective equipment was infrequently and heterogeneously used (e.g., nose clips vs. masks), and adherence was not measured. Third, collinearity with stronger acute exposures may have masked associations: training frequency and water temperature, both more proximate determinants of chloramine exposures, showed clear effects, whereas years federated and intensity may partly operate through these variables. Fourth, sample size constraints (*n* = 95) limit precision for modest effects and subgroup analyses, yielding wide confidence intervals. Finally, confounding by indication and reverse causality are possible: swimmers with more symptoms might preferentially use protective gear or avoid certain settings, biasing observed associations toward no effect. The non-significant trend for perceived air quality likely reflects subjective variability and the low sensitivity of self-report to ventilation performance (e.g., air-change rates, combined chlorine levels), which were not instrumentally measured.

It is important to clarify that AQUA is a screening tool rather than a diagnostic test, designed to identify athletes with symptoms compatible with allergic disease. A positive screen does not equate to a clinical diagnosis, and confirmation through objective testing (e.g., spirometry, IgE assays, skin prick tests) remains essential (23).

The strong associations with prior diagnosis and medication reinforce internal consistency between screening signals and care pathways, while the subset of AQUA-positive swimmers without a formal diagnosis suggests potential under-recognition. Incorporating standardized questionnaires such as AQUA into routine club-level screening could facilitate early referral to medical evaluation and optimize symptom control.

### 4.1 Strengths and limitations

This study provides novel data from a non-elite, federated population and uses a validated, sport-specific screening instrument (AQUA) with standardized, nationwide data collection. However, several limitations must be acknowledged. First, all variables were self-reported, making the data susceptible to recall bias and to exposure/outcome misclassification. Second, the online survey format may have introduced selection bias, as more motivated swimmers or symptom-aware individuals may have been more likely to participate. Third, AQUA positivity is a screening signal and does not equate to a confirmed clinical diagnosis; no objective physiological testing or clinical adjudication was performed. In addition, the cross-sectional design precludes causal inference; objective environmental monitoring (e.g., combined chlorine/trichloramine, air-exchange rates, temperature/humidity) was not conducted; the modest sample size limited precision and subgroup power; residual confounding and reverse causality cannot be excluded; and genetic susceptibility was not assessed.

Seasonal variation may also have influenced symptom reporting, as the survey was conducted during April–May, a period that coincides with peak pollen exposure in Portugal and may exacerbate allergic symptoms (33).

### 4.2 Implications and future directions

Findings support systematic screening in federated contexts and highlight environmental management of pools—particularly ventilation and water-temperature control—as actionable targets to mitigate symptom burden. Beyond elite settings, there is a clear need for structured prevention and monitoring protocols at the level of community and federated clubs. The national federation could play a pivotal role by promoting routine AQUA-based screenings, issuing guidelines for environmental standards in training facilities, and establishing referral pathways for athletes with suspected allergic disease. Interdisciplinary collaboration is essential to address this multifactorial issue. Physicians, sports scientists, environmental engineers, and public health professionals must work together to develop integrated strategies for symptom prevention, environmental control, and athlete education (34, 35).

Future studies should combine AQUA screening with objective physiological testing and comprehensive instrumental environmental monitoring—such as measurements of combined chlorine, trichloramine concentration, relative humidity, and air-exchange rates—while also incorporating detailed time-activity data and considering genetic susceptibility markers to refine risk stratification.

## 5 Conclusion

This study demonstrated a high prevalence of allergy-related symptoms among federated swimmers, with more than four out of five athletes screening positive on the AQUA questionnaire. Symptom burden was strongly associated with female sex, younger age, higher training frequency, and warmer water temperatures. These findings highlight the importance of systematic screening in federated swimming contexts and support the use of validated tools such as AQUA for early identification of athletes at risk.

The results also emphasize the role of pool environmental management, particularly ventilation and water temperature control, in mitigating respiratory and allergic morbidity. Implementation of structured screening protocols in clubs and federations may enable timely medical referral, optimize treatment, and minimize the impact of allergic disease on both performance and overall health. Future studies combining clinical evaluation with environmental monitoring are warranted to consolidate these findings and guide preventive strategies.

## Data Availability

The raw data supporting the conclusions of this article will be made available by the authors, without undue reservation.
