# Supplementary material for: Allergy-compatible symptoms among federated swimmers in Portugal: a cross-sectional study using the AQUA® questionnaire
Source: Front Public Health. 2026 Jan 12;13:1731628. doi: 10.3389/fpubh.2025.1731628 (PMC12832999; doi:10.3389/fpubh.2025.1731628)
Supplement: Supplementary file 1 [file Data_Sheet_1.DOCX]

**Allergy-compatible symptoms among federated swimmers in Portugal: a cross-sectional study using the AQUA® questionnaire**

**Supplementary material**

**Table S1** – Multivariable logistic regression model for predictors of AQUA©-positive screening among Portuguese federated swimmers (n = 95).

| Variable | B | S.E. | Wald | Sig. | OR | IC 95%  (Min) | IC 95%  (Max) |
| --- | --- | --- | --- | --- | --- | --- | --- |
| Nº of Training Sessions | 0.682 | 0.445 | 2.346 | 0.126 | 1.977 | 0.826 | 4.73 |
| Water Temperature | 1.18 | 0.748 | 2.49 | 0.115 | 3.254 | 0.751 | 14.09 |
| Age Range (21-25) | 1.809 | 1.285 | 1.981 | 0.159 | 6.106 | 0.492 | 75.831 |
| Sex (Male) | 0.167 | 1.017 | 1.91 | 0.167 | 0.245 | 0.033 | 17.99 |
| Anti-allergy medications | 0.995 | 6576.468 | 0.0 | 0.996 | 1.341e+16 | 0.0 | . |

Constant: B = -21.667 | Sig. = 0.996

Hosmer and Lemeshow: χ²(6) = 0.885, p = 0.990

Correct global classification: 88.4%

R² de Nagelkerke = 0.637

This table presents the full logistic regression output evaluating independent predictors of a positive AQUA© score (≥5 points). Coefficients (B), standard errors (SE), Wald statistics, p-values, adjusted odds ratios (OR), and 95% confidence intervals (CI) are provided for each predictor. Variance Inflation Factor (VIF) values are reported to document absence of problematic multicollinearity (all VIF < 2). Model diagnostics include Hosmer–Lemeshow goodness-of-fit, Nagelkerke R², and overall classification accuracy.

**Table S2** – Effect size measures (Cramer’s V and Cohen’s d) for bivariate associations between AQUA© status and demographic, training, and environmental variables.

| Variable | Cramer´s V |
| --- | --- |
| Sex | 0.281 |
| Age Range | 0.380 |
| BMI | 0.232 |
| Water Temperature | 0.113 |
| Air Quality | 0.198 |
| Frequency of Shortness of Breath | 0.215 |
| Use of Equipment | 0.018 |
| Training Intensity | 0.206 |
| Number of Training Sessions/Week | 0.314 |
| Years as Federated Swimmer | 0.149 |
| Previous Diagnosis of Allergic Disease | 0.313 |
| Use of Antiallergic Medication | 0.386 |
| Family History of Allergic Disease | 0.176 |

BMI= Body mass index

Effect sizes were reported using Cramer’s V. Values of approximately 0.10, 0.30, and 0.50 were interpreted as small, medium, and large associations, respectively. Cramer’s V values ranged from 0.02 to 0.39, indicating predominantly small to moderate associations between allergic status and the categorical predictor variables.

**Table S3** – Variance Inflation Factor.

| Variable | VIF |
| --- | --- |
| Sex | 1.334 |
| Nº of Training Sessions | 1.789 |
| Training Intensity | 1.519 |
| Water Temperature | 1.099 |
| Age Range | 1.824 |
| Anti-allergy medications | 1.234 |
| Family Allergy | 1.193 |
| Years Federated | 1.303 |


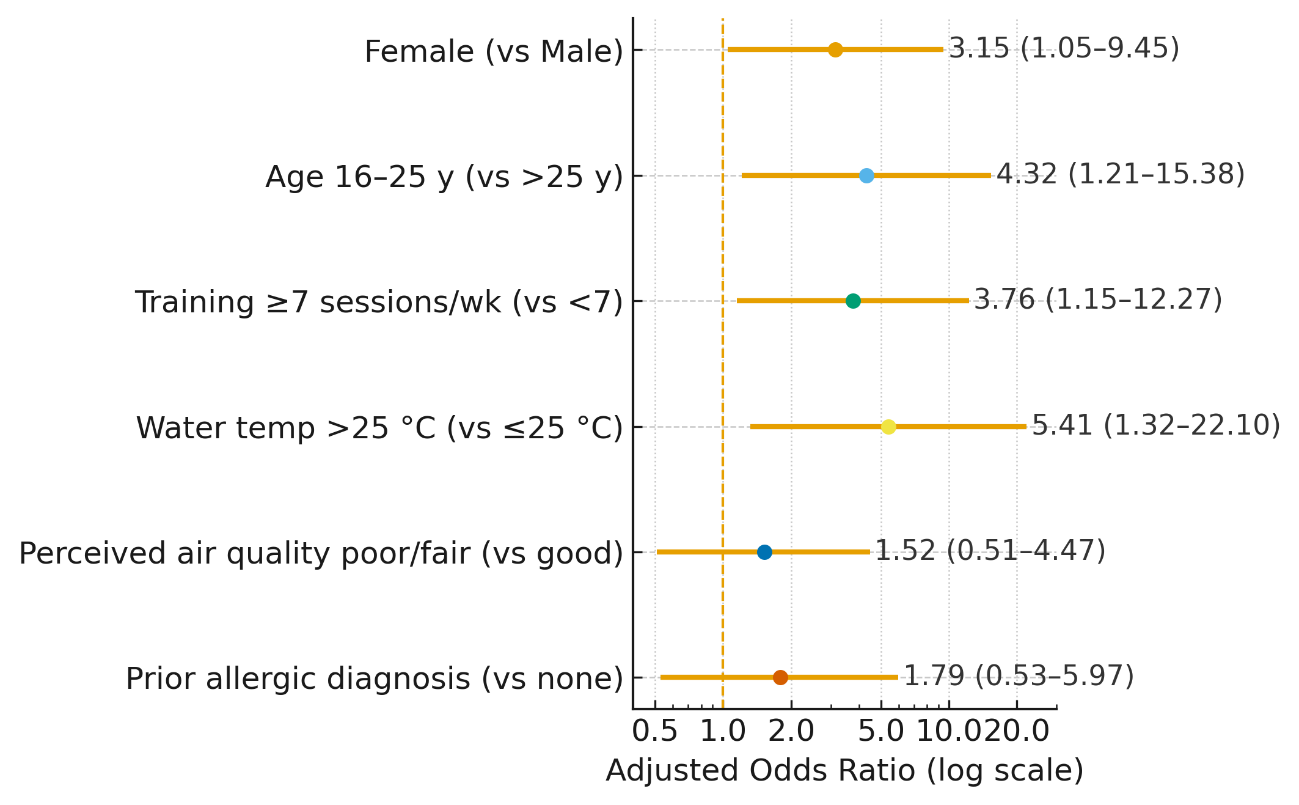


**Figure S1** – Forest plot displaying adjusted odds ratios (OR) and 95% confidence intervals (CI) from the multivariable logistic regression model assessing predictors of AQUA©-positive screening among Portuguese federated swimmers (n = 95).

The figure presents the independent associations between demographic, training, environmental, and clinical variables and the likelihood of screening positive on the AQUA© questionnaire (score ≥5). Each point represents an adjusted OR, with horizontal lines indicating corresponding 95% CIs. A vertical reference line at OR = 1 denotes the point of no association. The model identified female sex, younger age (16–25 years), high training volume (≥7 sessions/week), and pool water temperature >25 °C as statistically significant predictors. Variables with confidence intervals crossing 1 were not significantly associated with AQUA© positivity. Full regression coefficients, standard errors, p-values, and VIF values are provided in Supplementary Table S1.
